# Beneficial roles of probiotics on the modulation of gut microbiota and immune response in pigs

**DOI:** 10.1371/journal.pone.0220843

**Published:** 2019-08-28

**Authors:** Donghyun Shin, Sung Yong Chang, Paul Bogere, KyeongHye Won, Jae-Young Choi, Yeon-Jae Choi, Hak Kyo Lee, Jin Hur, Byung-Yong Park, Younghoon Kim, Jaeyoung Heo

**Affiliations:** 1 Department of Animal Biotechnology, Chonbuk National University, Jeonju, Republic of Korea; 2 Department of Animal Science and Institute of Milk Genomics, Chonbuk National University, Jeonju, Republic of Korea; 3 Department of Agricultural Convergence Technology, Chonbuk National University, Jeonju, Republic of Korea; 4 The Animal Molecular Genetics and Breeding Center, Chonbuk National University, Jeonju, Republic of Korea; 5 International Agricultural Development and Cooperation Center, Chonbuk National University, Jeonju, Republic of Korea; 6 College of Veterinary Medicine, Chonbuk National University, Iksan, Republic of Korea; 7 Department of Agricultural Biotechnology and Research Institute of Agriculture and Life Science, Seoul National University, Seoul, Republic of Korea; Institute of Technology of Agricultural Products, GREECE

## Abstract

The importance of probiotics in swine production is widely acknowledged as crucial. However, gaps still remain in the exact roles played by probiotics in modulation of gut microbiota and immune response. This study determined the roles of probiotic *Lactobacillus plantarum* strain JDFM LP11in gut microbiota modulation and immune response in weaned piglets. *L*. *plantarum* JDFM LP11 increased the population of lactic acid bacteria in feces and enhanced the development of villi in the small intestine. Metagenome analysis showed that microbial diversity and richness (Simpson, Shannon, ACE, Chao1) and the relative abundance of the Firmicutes were higher in weaned piglets fed probiotics. Five bacterial families were different in the relative abundance, especially; *Prevotellaceae* occupied the largest part of microbial community showed the most difference between two groups. Transcriptome analysis identified 25 differentially expressed genes using RNA-sequencing data of the ileum. Further gene ontology and immune DB analysis determined 8 genes associated with innate defense response and cytokine production. BPI, RSAD2, SLPI, LUM, OLFM4, DMBT1 and C6 genes were down-regulated by probiotic supplementation except PLA2G2A. PICRUSt analysis predicting functional profiling of microbial communities indicated branched amino acid biosynthesis and butyrate metabolism promoting gut development and health were increased by probiotics. Altogether, our data suggest that *L*. *plantarum* JDFM LP11 increases the diversity and richness in the microbial community, and attenuates the ileal immune gene expression towards gut inflammation, promoting intestinal development in weaned piglets.

## Introduction

Animal productivity and health are affected by various factors such as nutrition, environment, or even dietary changes. In swine production, piglets are severely infulenced by stress that ensues after weaning and leads to major economic losses to swine farmers [1]. Devastating symptoms after weaning include diarrhea, reduced feed conversion efficiency, loss in weight and in extreme cases death [2]. Because of many losses associated with post weaning diarrhea, antibiotic feed additives had for so long been used as therapeutic alternatives and growth promoters [3]. However, due to the increasing antibiotic resistances in intestinal microbes to antibacterial drugs, and the associated transfer of the same resistance to pork consumers coupled with bans to the use of these antibiotics in food, farmers have sought better alternatives [3, 4]. Probiotics have been suggested and used as better alternatives to antibiotic use as remedy to post weaning diarrhea and as growth promoters [5, 6]. FAO and WHO have defined probiotics as live microorganisms administered in sufficient amounts to confer health benefits to the host [7]. However, the term probiotic being generic in nature has also been extended to include organisms such as yeast cells, bacteria cells, or a combination of the two which act to manipulate the gastrointestinal environment so as to improve the health of the host [8]. Various bacteria have been used as probiotics however, *Lactobacillus* is the most widely used probiotic agent. Bacteria including *Bifidobacterium*, and yeast *Saccharomyces boulardii* have also been used to confer probiotic effects to swine and other hosts [9, 10]. Studies have documented the effects of probiotic supplementation in swine diets including improvement in growth performance, feed conversion efficiency, intestinal microbiota modulation, nutrient utilization, gut health, and regulation of the immune system [1, 11, 12]. Additionally, probiotics have been demonstrated to have anti-infectious properties like reduction of colonization and shedding of *Salmonella*, and reducing post weaning Colibacillosis due to Enterotoxigenic *Escherichia coli* (ETEC) [13, 14]. Probiotics have been recognized to affect their hosts through various mechanisms of action such as gut microbial manipulation, competition for adhesion sites on the mucosa, strengthening gut epithelial barrier function, and regulating the immune system [9, 15, 16], and these mechanisms involve expression of genes in specific tissues mostly the intestines and the liver [17].

Probiotics, for example, *Lactobacillus salivarius* UC118 and *Enterococcus faecium* NCIMB 11181 have been studied and found to have positive effects on gut microbiota through stabilizing the bacterial community of piglets as evidenced by the increased bacterial diversity and richness [18, 19]. Another study by [20] demonstrated that probiotic supplementation in weaned piglets improved intestinal microbial balance, immunity and overall growth performance. However, despite the many studies indicating the role of probiotics in animals, assessing the effect of the same on the host gut microbial community has become a focus of research in the past years [21]. The need to assess probiotics’ impact on host gut microbiota is further emphasized due to the fact that probiotics could lead to certain metabolic disorders in the host [18, 19]. Piglets are confronted with a big challenge to develop their immune system at weaning stage which should adapt to GI microbial colonization and antigens from milk or feed [22]. However, gaps still remain in the exact roles played by probiotics in modulation of piglet microbiota and immune response. In this study, we investigated beneficial roles of probiotics in gut microbiota modulation and immune response on gut heath in three-way crossbred (LYD) weaned piglets through analysis of fecal microbiome and intestinal transcriptome using *L*. *plantarum* strain JDFM LP11 possessing significant probiotic potential [23] and influencing on meat quality and chemical characteristics in longissimus muscles of slaughtered pigs [24].

## Materials and methods

### Ethics statement

All animal experiments were performed with in accordance with national and university guidelines. The animal protocol reported in this study was approved by the Chonbuk National University Animal Ethics Committee in accordance with the guidelines of the Korean Council on Animal care (CBNU 2015–029).

### Animals, diets and probiotics treatment

In order to evaluate the beneficial roles of liquid probiotics on the modulation of gut microbiota and gene expression in young pig, a total of 6 female three-way crossbred (Landrace x Yorkshire x Duroc) piglets were reared on a conventional farm (Dowon farm, Jeolabuk-do, South Korea) or on the liquid probiotics application system farm as we described previously (Doozy farm, Jeolabuk-do, South Korea) [24] (3 piglets per farm). The probiotic farm provided both liquid and solid probiotics to the sows and piglets via drinking and feeds. At 4 weeks after birth, piglets were weaned and transferred to the experimental swine unit of College of Veterinary Medicine at Chonbuk National University as a way to minimize environmental effects such as housing and management measures between the farms. The two groups were fed a similar ration of feed with the addition of liquid probiotics to the piglets from the probiotic farm, hence piglets from the conventional farm were designated as controls. Each group was housed in separate but under the same conditions to prevent cross-contamination between groups. Each room was equipped with ventilation fan and separate air-conditioning system. Liquid probiotics used in this study containing *L*. *plantarum* strain JDFM LP11 (2.5×10^7^ CFU/ml), 1% glucose, 1% molasses, 0.2% sea salt and 0.2% yeast extract (Eco Probiotics Solution, lot number: 061217, Doozy Probiotics Co., Ltd., Jeolabuk-do, South Korea) as described previously [23, 24] were stored at 4 °C and mixed into a weaner diet (FARMSCO Inc., Gyeonggi-do, South Korea) every morning for probiotic group (50 ml aliquots/kg of diet, 1.25×10^9^ CFU/kg of diet). After 1 week allowing for adaptation, each piglet was fed a weaner diet or probiotic diet *ad libitum* with free access to fresh water during a 4 week trial.

### Blood sampling and serum immunoglobulin G assay

Blood samples were collected on days 0, 14 and 28 for measurement of serum immunoglobulin G (IgG) during a 4 week trial. Whole blood samples were collected in serum collection tubes (BD Vacutainer SSTTM II Advance, Becton Dickinson, Plymouth, UK) from the jugular vein of pigs and centrifuged at 2500×g for 20 min within 3 h to obtain serum. Serum was stored at –20°C for subsequent analysis of IgG antibody. To investigate IgG concentrations in serum, standard ELISA was performed using the pig IgG ELISA quantitation kit according to the manufacturer’s instructions (Bethyl Lab, Montgomery, TX, USA).

### Collection of rectal feces and intestinal tissues

At the end of animal study, all piglets were euthanized by being administered intramuscularly with ZoletilTM 50 (Virbac; 7–10 mg/kg of body weight; Carros, Cedex, France) and xylazine (2.32–3.48 mg/kg; Bayer Korea, Ansan, South Korea) for sampling. Sterile tubes were used to collect rectal fecal samples from each piglet, immediately frozen by dry ice and stored at -80°C for subsequent fecal microbiota DNA isolation. The small intestine was removed from the cavity and divided into 10 parts of equal length. The distal part of the small intestine (segment 8) were collected in a sterile tube and immediately frozen by dry ice and stored at -80 °C for subsequent RNA isolation.

### Histochemical staining

The small intestine (duodenum, jejunum and ileum) of experimental piglets were collected and fixed with 10% neutral buffered formalin (NBF). Paraffin embedded tissue block were sectioned at 5 μm. Sections were de-paraffinized in xylene and hydrated in a descending series of ethanol and then stained with hematoxylin and eosin (HE) for examining the histological organization of the tissues. Stained sections were dehydrated in an ascending series of ethanol, cleared in xylene and mounted on slide. Digital images were acquired using a Leica DM2500 microscope (Leica Microsystems, Germany) at fixed 100 x magnification.

### Enumeration of lactic acid bacteria

10 g of fecal samples were aseptically removed from the sterile tubes and placed into whirl-pak bags containing 90ml of 0.1% peptone water. Samples were then stomached for 2 min and serial dilutions were made. Diluted samples were then plated on de Man, Rogosa & Sharpe (MRS) plates containing 0.05% (w/v) bromocresol purple (BCP). The plates were then incubated anaerobically at 37°C for 48h. Counts were recorded as colony forming units per gram (cfu/g).

### Fecal DNA preparation and microbial community analysis

**Fig 1:**
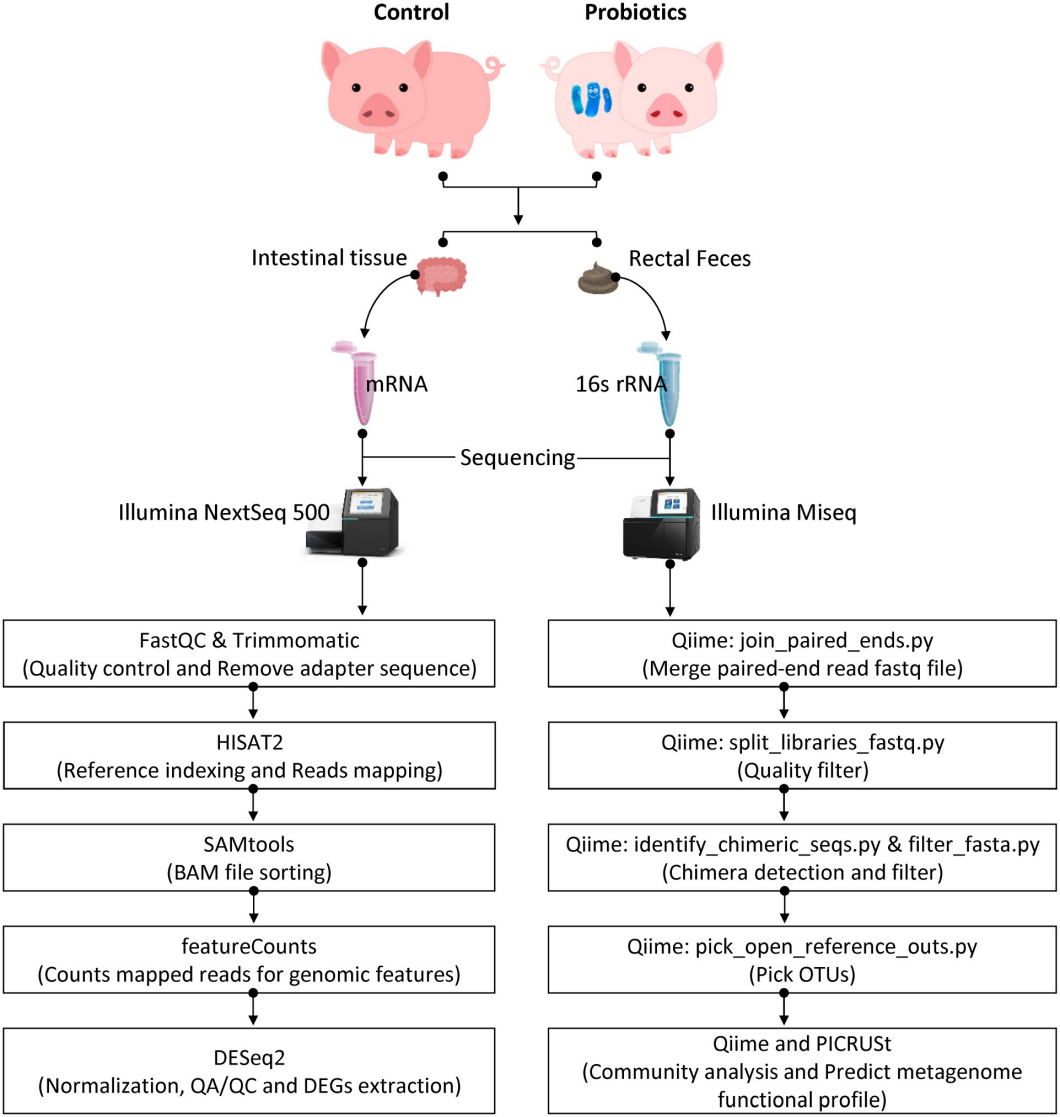
Flow diagram showing experimental paradigm through analyses of fecal microbiome and intestinal transcriptome. Female piglets (5 weeks old) were fed a weaner diet or a weaner diet mixed with liquid probiotics containing *L*. *plantarum* strain JDFM LP11 (1.25×10^9^ CFU/kg of diet) for 4 weeks (n = 3 per group). Rectal feces and ileal tissues were collected at the end of animal trial for analyses of fecal microbiome and intestinal transcriptome to study roles of probiotics in modulation of gut microbiota and immune associated gene expression in weaned piglets.

Flow diagram showing experimental paradigm through analysis of fecal microbiome and intestinal transcriptome in weaned piglets was illustrated in Fig 1. DNA was isolated using Epicentre DNA isolation kits (3 biological replicates in each dietary group). Approximately 900 ng of DNA were extracted from each sample. DNA quality was confirmed by a Bioanalyzer using an Agilent RNA 6000 Pico Kit (Agilent, Santa Clara, CA). All the samples from the reservoir were prepared using the 16S library preparation protocol and the Nextera XT DNA index kit (illumina, San Diego, CA) to target the V3-V4 variable regions of the 16S rRNA gene. Quantification of library was measured by real-time PCR using CFX96 real time system (BioRad, Hercules, CA). All samples passed a QC test. Samples were loaded onto a MiSeq reagent catridge (illumina, San Diego, CA) and then onto the instrument. Automated cluster generation was done and a 2x300bp paired-end sequencing were performed. The resulting sequence reads were equally distributed across the samples.

The Illumina MiSeq technology can generate up to 10^7^ sequences in a single run [25]. The Quantitative insights into microbial ecology, QIIME (1.9.1 version) [26] then takes the instrument output and generates useful information about the community in each sample. We divided the process into upstream and downstream stages. The sample identifier, barcode and primer sequence information were required for the upstream stage of the QIIME workflow. This processing step combines sample demultiplexing, primer removal and quality filtering. The first step in the upstream stage was to merge paired-end Illumina reads using ‘joint_paired_ends.py’ script. We then performed demultiplexing of merged fasta sequence data using ‘split_libraries_fastq.py’ script with—barcode_type ‘not-barcoded’ option because our sequence data already removed barcode sequence. During the PCR amplification process, some of the amplified sequences can be produced from multiple parent sequences, generating sequences known as chimeras. Therefore, we identified chimeric sequences in fasta files from GREENGENES database [27] using ‘identify_chimeric_seqs.py’ script and vsearch (2.4.4 version) [28]. And then we removed identified chimera sequences from fasta files using ‘filter_fasta.py’ script. The next step is clustering the preprocessed sequences into Operational Taxonomic Units (OTUs), which in traditional taxonomy represent groups of organisms defined by intrinsic phenotypic similarity that constitute candidate taxa [29, 30]. For DNA sequence data, these clusters, and hence the OTUs, are formed based on sequence identity. In other words, sequences are clustered together if they are more similar than a user-defined identity threshold, presented as a percentage (s). This level of threshold is traditionally set at 97% of sequence similarity, conventionally assumed to represent bacterial species [31]. Open-reference OTU picking process was carried out using ‘pick_open_reference_otu.py’ script that reads are clustered against a reference sequence collection and any reads which do not hit the reference sequence collection are subsequently clustered de novo [32]. In downstream stage, diversity (Shannon and Simpson) and richness (Observed, Chao1 and ACE) [32–34] analysis of control and probiotics groups using ‘alpha_diversity.py’ and ‘estimate_observation_richness.py’ script was done. Microbial communities were compared based on their compositional structures. Unweighted and weighted UniFrac analysis of control and probiotics groups using QIIME was performed. Multi-level taxonomic abundance was extracted using QIIME and Student’s t-test was used to detect differentially abundant microbiota by comparing relative abundance between the control and probiotics groups. For consideration of different read production, proportion was used instead of read count.

### RNA preparation and RNA-seq analysis

RNA was isolated from the ileum tissues using the TRIzol reagent (Invitrogen) based on the manufacturer instructions. The quality of RNA samples was verified using the absorbance ratio (2.08–2.10) at 260 nm/280 nm by a NanoDrop 1000 spectrophotometer (Thermo Scientific, Wilmington, DE, USA), and by RNA integrity number (RIN) score of above 7 as analyzed on a Bioanalyzer 2100 (Agilent technologies, Palo Alto, CA, USA). The mRNA in total RNA was converted into a library of template molecules suitable for subsequent cluster generation using the reagents provided in the Illumina TruSeq RNA Sample Preparation Kit. In summary, mRNA was purified using poly-A selection, then chemically fragmented and converted into single-stranded cDNA using random hexamer priming. The second strand is then generated to create double-stranded cDNA that is ready for TruSeq library construction. The short ds-cDNA fragments were then connected with sequencing adapters, and suitable fragments were separated by agarose gel electrophoresis. Finally, truseq RNA libraries were built by PCR amplification, quantified using qPCR according to the qPCR Quantification Protocol Guide, qualified using the Agilent Technologies 2100 Bioanalyzer. (Agilent technologies,Palo Alto CA, USA). Based on the generated RNA libraries, paired-end sequencing (101 bp read-length and approximately 150 to 180 insert size) was performed using the HiSeq NextSeq 500 platform (Illumina,San Diego, USA). Next, to measure transcriptome levels with generated RNA-seq reads we performed the following widely used RNA-seq pipeline: (1) We employed Trimmomatic (v0.32) [35] with following option: PE -phred33 ILLUMINACLIP:TruSeq3-PE.fa:2:30:10 LEADING:3 TRAILING:3 SLIDINGWINDOW:4:15 MINLEN:36 for making clean reads. (2) We mapped such clean reads into genome reference (Sscrofa11.1) from Ensemble database using hisat2 (v2.1.0) [36]. (3) We used the featureCounts in SUBREAD packages (v 1.6.0) [37] to estimate the count of uniquely mapped reads for each of the 25,882 annotated genes in the Sus scrofa gene transfer format (GTF) file. From this RNA-seq analysis pipeline, we obtained the transcriptome expression level of 25,880 genes from 6 samples.

The DESeq2 package was employed to distinguish differentially expressed genes (DEGs) between the control and probiotics groups [38]. First, DESeq2 used empirical Bayes shrinkage method to estimate dispersions and fold changes by modeling read counts as following a Negative Binominal distribution. And then the Wald test P-value was inferred to evaluate the statistical significance. The estimated p-values were adjusted for multiple testing using FDR method to control the false positive due to numerous tested genes in a typical RNA-Seq dataset. Finally, the DEGs were declared at a significant level of base-mean >50, |log2 (fold change)| > 1.5 and FDR < 0.05. The pig Ensemble gene IDs were converted into official gene symbols by cross matching to pig and human Ensemble gene IDs. The official gene symbols of pig genes were then used for functional clustering and enrichment analyses using the WebGestalt [39]. The representation of functional group in transcriptome comparison between the control and probiotics groups was investigated using the gene ontology (biological process). The gene ontology of DEGs was carried out using overrepresentation enrichment analysis (ORA) method and gene ontology database.

### Quantitative real-time PCR analysis for DEGs

To synthesize cDNA was using the ReverTra Ace (Toyobo, Osaka, Japan) according to manufacturer’s guidelines. Real-time qPCR was using the iTaq Universal SYBR Green Supermix (Bio Rad, Hercules, CA, USA) according to manufacturer’s guidelines and the solution was constructed as follows: 1 μL diluted cDNA was added to 5 μL iTaq Universal SYBR Green Supermix, 1 μL each of 5 pmol/μL diluted forward and reverse primers. C1000 Thermal Cycler (Bio Rad, Hercules, CA, USA) to measure the expression of target gene. Conditions used for real-time qPCR were as follows: incubation at 95°C for 5 min followed by 40 cycles of denaturation at 95°C for 10 s and annealing at 64°C for 30 s. All measurements were performed 3 times for each sample and relative gene expression was using the 2–ΔΔCt method [40]. Relative expression of the target gene was normalized to Glyceraldehyde 3-phosphate de12hydrogenase (GAPDH). The expression of DEGs was conducted by qRT-PCR using the primers listed in S7 Table.

## Results

### Lactic acid bacteria

The *L*. *plantarum* strain JDFM LP11 used in this study possesses significant probiotic potential, with enhanced acid/bile tolerance, attachment to porcine intestinal epithelial cells (IPEC-J2), and antimicrobial activity [23]. Average daily intake of *L*. *plantarum* JDFM LP11 in the probiotics group was 1.2×10^9^ CFU/pig during a 4 week trial. Plate counts were performed to establish the viability in LAB in both the control and probiotics groups. We determined that the number of LAB in fecal samples was significantly higher in the probiotics group compared to the control at p<0.05 (S1 Fig).

### Effect of probiotics on the gut epithelial layer

**Fig 2:**
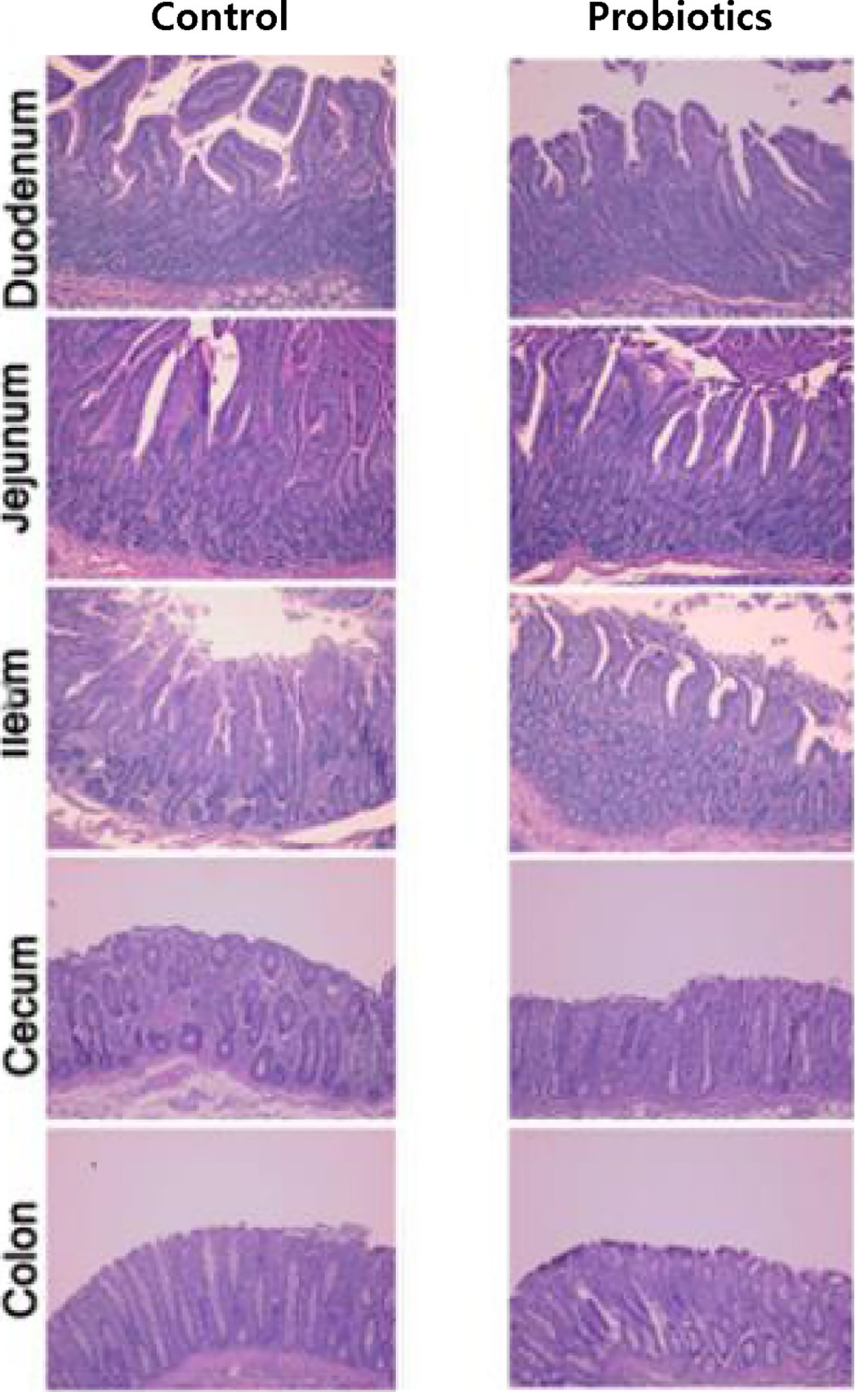
Photomicrographs of epithelial layers of the small intestine, cecum and colon in piglets between the control and probiotics groups. The villus height and crypt depth in intestinal segments were increased by probiotic supplementation.

Photomicrographs of epithelial layers of the small intestine, caecum, and colon revealed increases in the villus height and crypt depth in the probiotics treated piglets (Fig 2). The villus heights in segments of duodenum, jejunum, and ileum were greatly increased with probiotic supplementation.

### Gut microbial diversity

**Fig 3:**
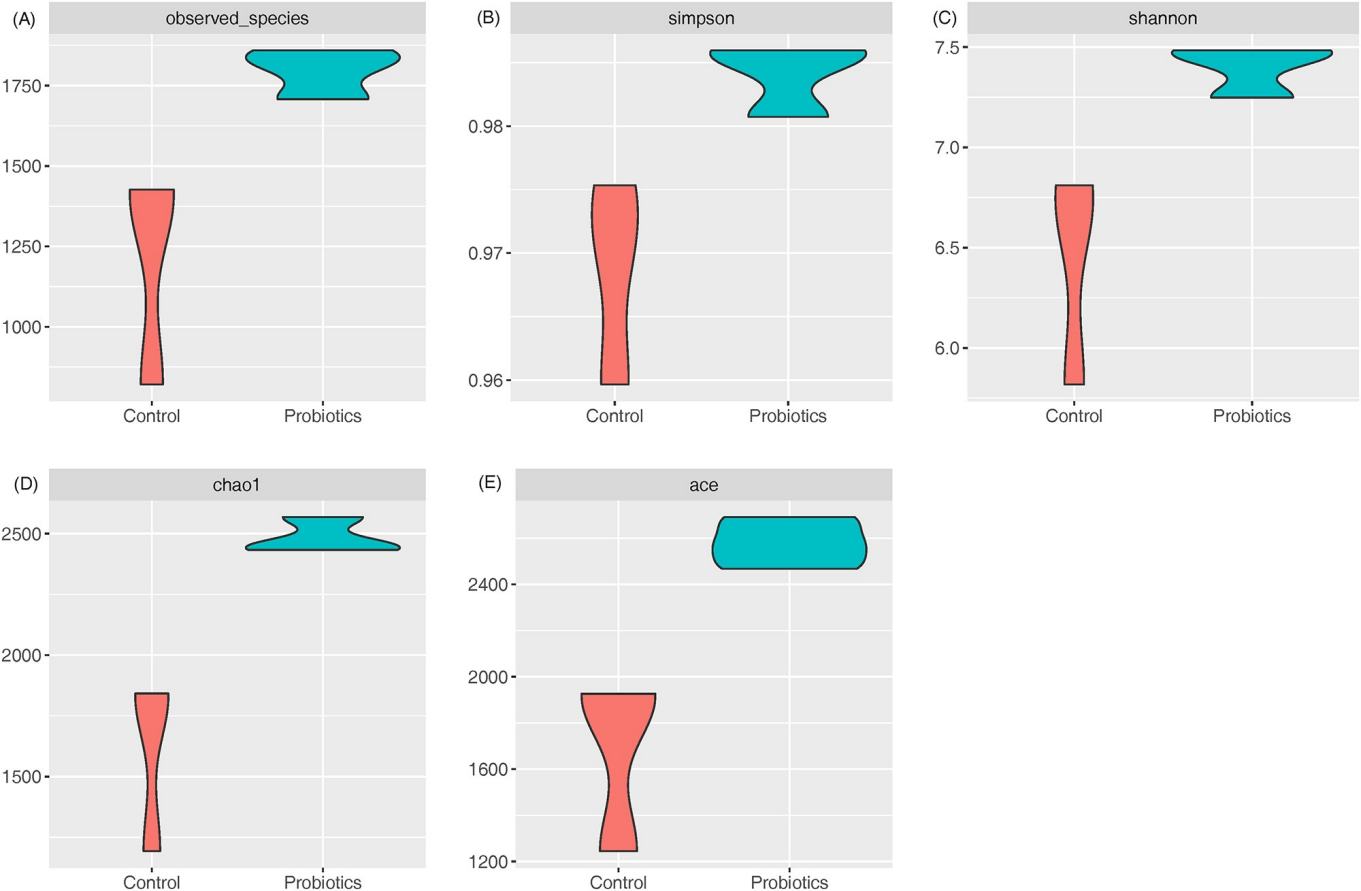
Microbial diversity and richness in fecal samples of piglets between the control and probiotics groups. (A) Observed index; (B) Simpson index; (C) Shannon index; (D) Chao1 index; (E) ACE index. Differences are p<0.05 as measured by T-test. Alpha-diversity analyses indicate probiotic supplementation increased the microbial diversity and richness.

**Fig 4:**
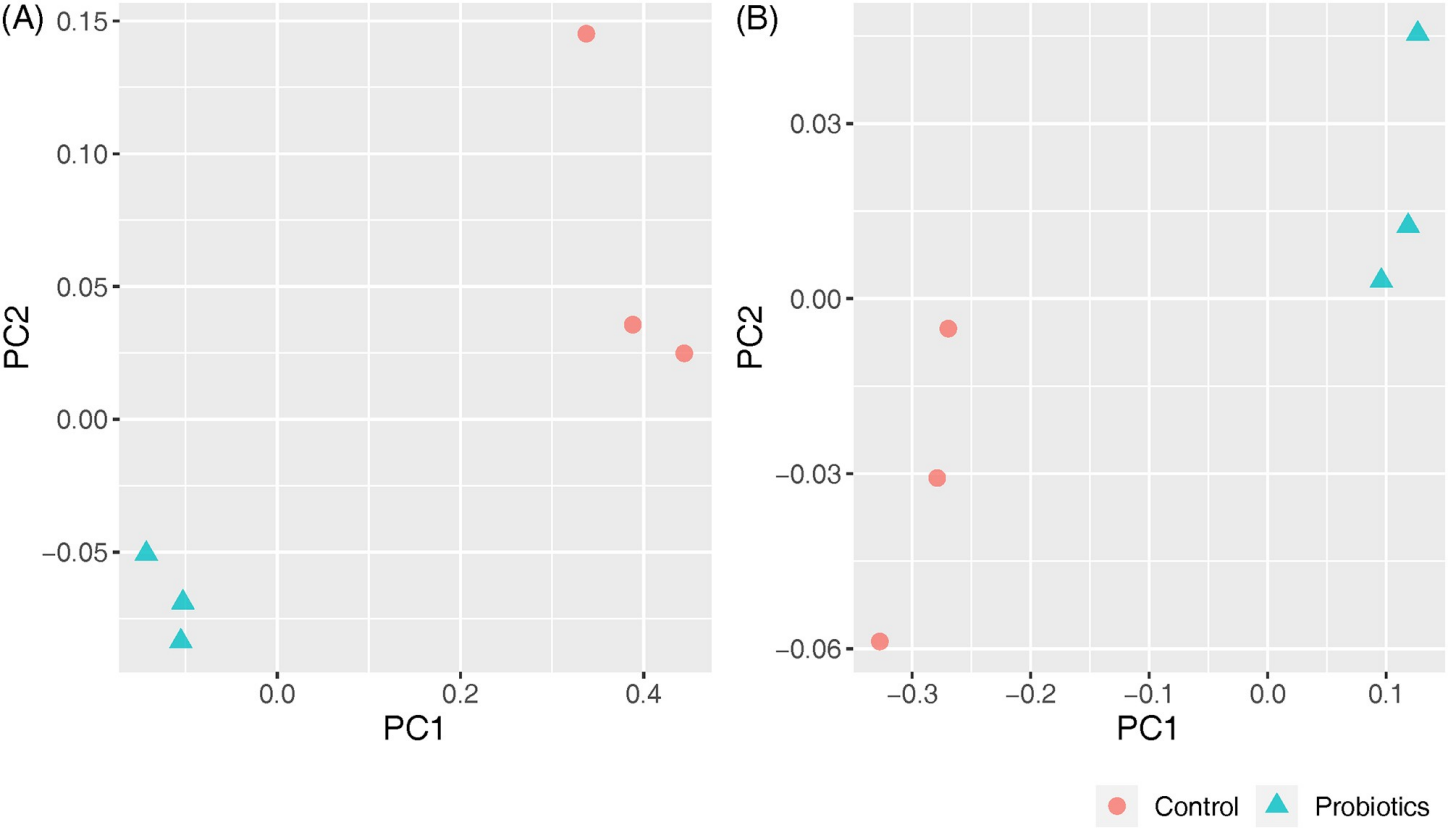
Principal coordinates analysis (PCoA) plots in fecal samples of piglets between the control and probiotics groups. (A) Unweighted UniFrac analysis. (B) Weighted UniFrac analysis. Each point represents an individual piglet. The probiotics group was clearly separated from the control group microbiota in both unweighted and weighted UniFrac analyses. These data indicate that probiotics supplementation changes the shape of fecal microbial community in piglets.

16S rRNA gene sequencing was used to monitor changes in the microbial community in the fecal samples. Alpha-diversity analyses revealed that probiotic supplementation increased the microbial diversity and richness (Fig 3). The probiotics group was significantly higher than the control group in the number of microbial species (Fig 3: Observed, Chao1 and ACE). The explicitly model evenness suggested that the probiotics group was significantly higher than the control group in the microbial diversity (Fig 3: Shannon and Simpson). Beta-diversity result measured by principal coordinates analysis (PCoA)indicated the probiotics group was clearly separated from the control group microbiota in both unweighted and weighted UniFrac analyses (Fig 4).

### Taxonomic composition comparison

In rarefaction curve data of this study, we first determined operational taxonomic units (OTUs) of each sample at species level. As shown in under rarefaction curve figure (S6 Fig), the number of OTUs in the probiotics group was higher than those in the control group. Based on this result, we thought that probiotics led to alteration of the intestinal microbial community to normal. Although the number of one individual in the control group was lower than other two individuals in the same group, overall conclusion would not be affected.

16S rRNA sequencing revealed variabilities in the microbial composition and relative abundance at phylum level in fecal microbiota of piglets. Two most abundant phyla, Bacteroidetes and Firmicutes, were observed in both groups. However, the percentages of relative abundances of Bacteroidetes, Firmicutes and Spirochaetes were significantly different at p-values 0.003, 0.006 and 0.039, respectively, between the control and probiotics groups (S2 Fig and S1 Table).

Microbial taxonomy at order level shows that the orders, Bacteroidales and Clostridiales were the most abundant in both groups. The relative abundancies of the orders Bacteroidales, Clostridiales, Erysipelotrichales, Sphaerochaetales and Spirochaetales between two groups were significantly different with p-values 0.003, 0.005, 0.019, 0.023, and 0.04, respectively (S4 Fig and S3 Table).

**Fig 5:**
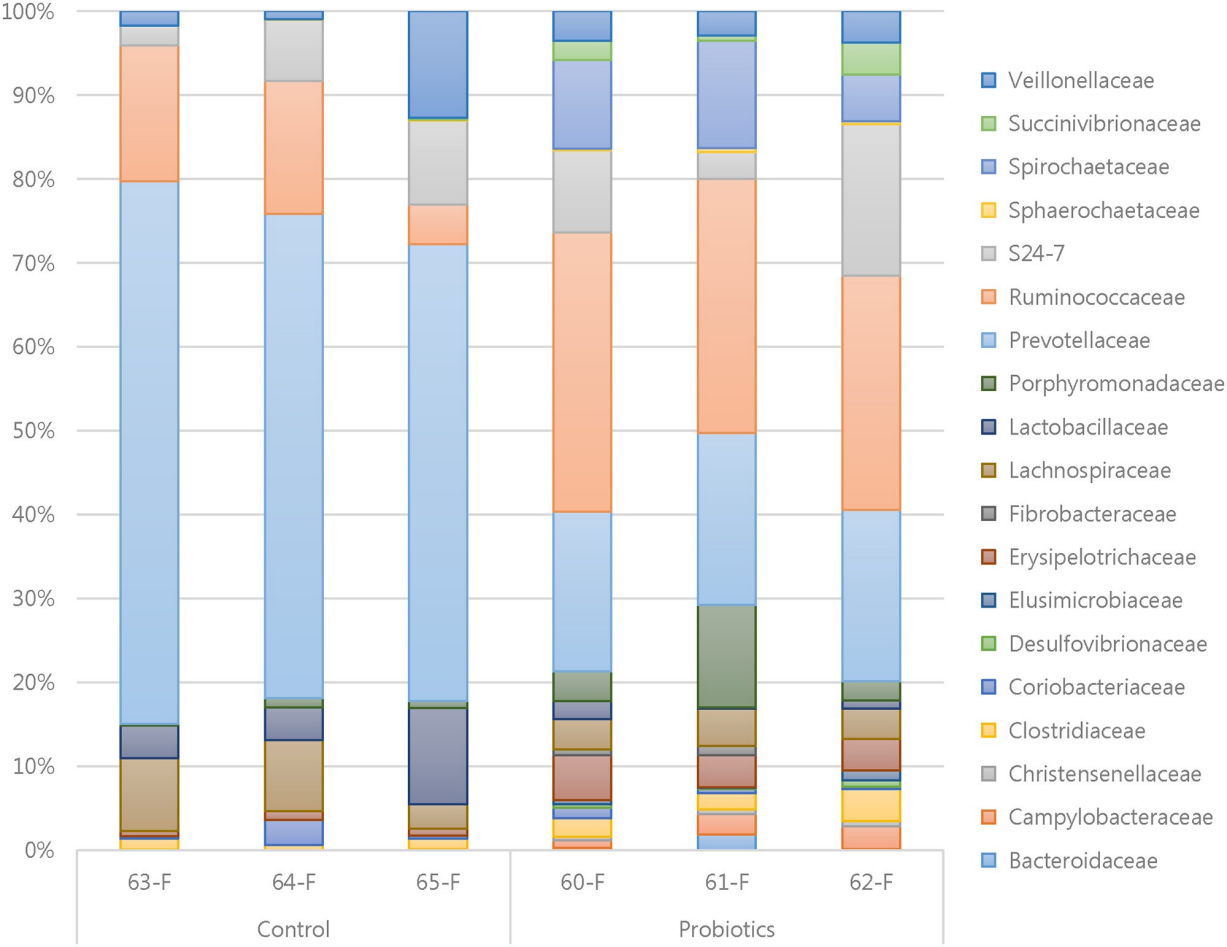
Histogram representing taxonomic composition and relative abundance (>0.1%) at family level in fecal samples between the control and probiotics groups. A total of 19 different families of bacteria were found in two groups. *Prevotellaceae* and *Ruminococcaceae* were the most abundant families.

A total of 19 different families of bacteria were found in two groups, and *Prevotellaceae* and *Ruminococcaceae* were the most abundant families (Fig 5). The families *Prevotellaceae*, *Erysipelotrichaceae*, *Sphaerochaetaceae*, *Spirochaetaceae* and *Christensenellaceae* were significantly different between two groups (p<0.05, S4 Table).

### Transcriptome analysis in the small intestine

**Fig 6:**
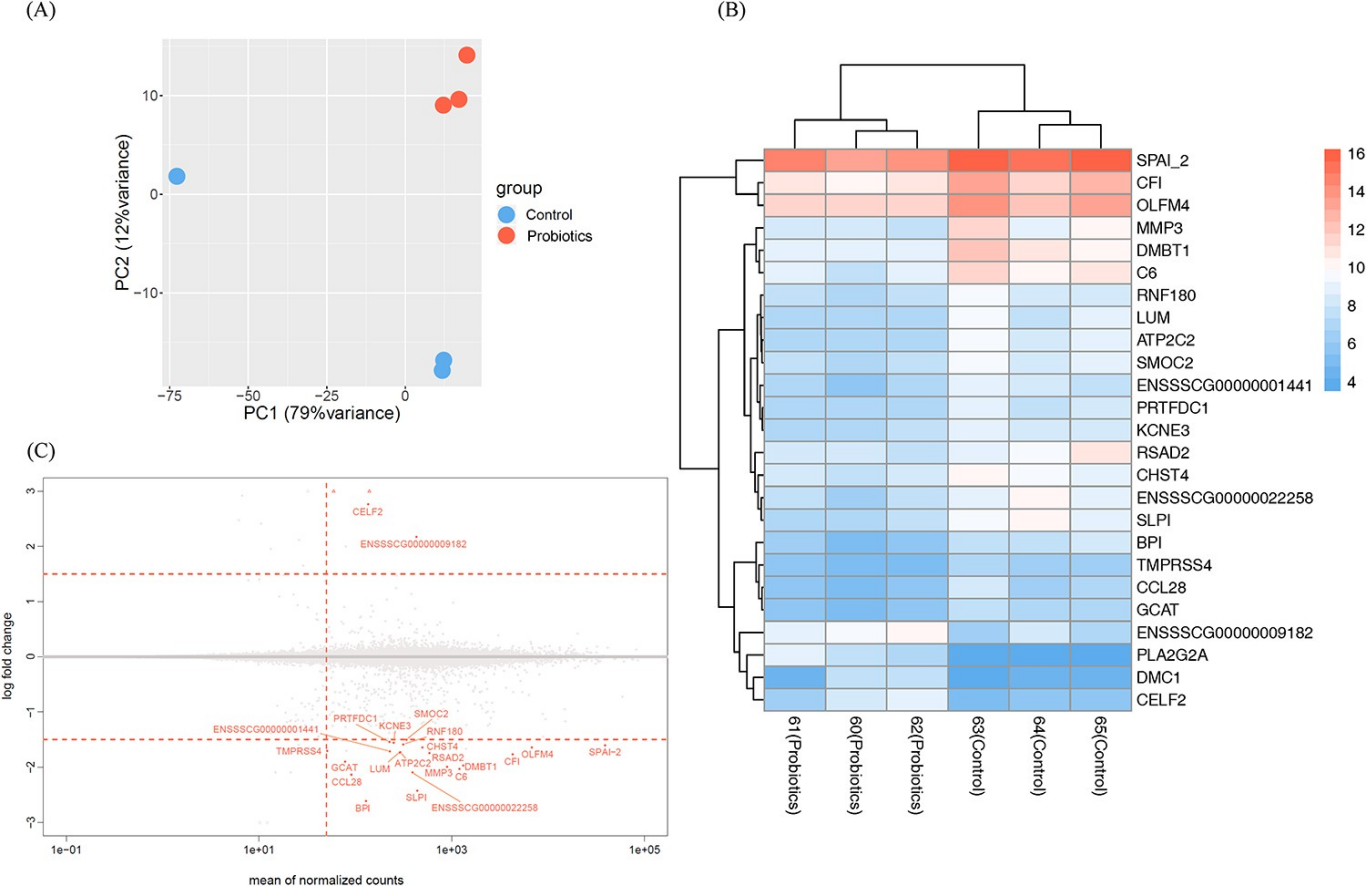
Expression pattern comparative analysis between the control and probiotics piglets. (A) Principal component analysis (PCA) plot between the control and probiotics RNA-seq samples. (B) Heatmap of DEGs distinguishes the control and probiotics piglets. The color scale represents a low expression value in blue and a high expression value in red. (C) Expression of DEGs between control and probiotics piglets.

We produced RNA-seq reads from the small intestine (ileum) of piglets in both groups which were deposited in the NCBI Gene Expression Omnibus. The quality report for RNA-seq revealed that average of passed sequencing quality criteria using the Trimmomatic tool was 95.15% and the average number of sequence reads was 16.6 and 15.9 million in the control and probiotics groups, respectively. In addition, most alignment rates for two groups exceeded 96%, which were mapped successfully to the pig reference genome (Sscrofa11.1) using Hisat2 [36]. The numbers of total sequence reads, read order, index, yield, and mapping rates for each sample are shown in S5 Table. Furthermore, a PCA plot, a heatmap of DEGs and a scatter plot (mean of normalized counts and log fold change of total genes) are presented in Fig 6.

Principal component analysis shows that readcount data of host expression clearly distinguished between the control and probiotics groups (Fig 6A). Using RNA-seq analysis, 25 DEGs were identified in the ileum of weaned piglets (S6 Table), and these DEGs were compared with each sample using heatmap visualization to confirm the expression pattern (Fig 6B). The expression of 22 genes was down-regulated by probiotic supplementation, while the expression of three genes was up-regulated (Fig 6C). These data indicate that probiotic supplementation gives a significant effect on the ileal gene expression pattern in weaned piglets.

We used WebGestalt to perform analysis of DEGs co-occurrence in the ileum of piglets by comparison of the control and probiotics groups, and identified 10 gene ontologies in the biological process range (Table 1). Through this analysis, we found that DEGs were mainly and significantly involved in immune response, because seven out of 10 gene ontologies were significantly associated with immune response (defense response to other organism, regulation of cytokine production, cytokine production, response to other organism, response to external biotic stimulus, response to biotic stimulus, and defense response to bacterium). Bactericidal/permeability-increasing protein (BPI), radical SAM domain-containing 2 (RSAD2) and secretory leukocyte protease inhibitor (SLPI) were relevant to defense response to other organism (GO:0098542), response to other organism (GO:0051707), response to external biotic stimulus (GO:0043207), and response to biotic stimulus (GO:0009607). BPI and SLPI were related to defense response to bacterium (GO:0042742), and BPI, RSAD2 and lumican (LUM) were associated with regulation of cytokine production (GO:0001817) and cytokine production (GO:0001816).

**Table 1:** Gene ontology (GO) analysis of differentially expressed genes (biological process).

GO ID	Description	#Genes	Enrichment	p-value	Genes
GO:0098542	Defense response to other organism	3	12.44	0.001	BPI, RSAD2, SLPI
GO:0001817	Regulation of cytokine production	3	11.54	0.002	LUM, BPI, RSAD2
GO:0001816	Cytokine production	3	10.57	0.002	LUM, BPI, RSAD2
GO:0051707	Response to other organism	3	7.90	0.005	BPI, RSAD2, SLPI
GO:0043207	Response to external biotic stimulus	3	7.87	0.005	BPI, RSAD2, SLPI
GO:0009607	Response to biotic stimulus	3	7.37	0.006	BPI, RSAD2, SLPI
GO:0042742	Defense response to bacterium	2	15.81	0.007	BPI, SLPI
GO:0010959	Regulation of metal ion transport	2	14.81	0.008	ATP2C2, KCNE3
GO:0010466	Negative regulation of peptidase activity	2	14.09	0.008	SLPI, SPAI-2
GO:0000730	DNA recombinase assembly	1	116.98	0.009	DMC1

BPI: Bactericidal/permeability-increasing protein, RSAD2: Radical SAM domain-containing 2, SLPI: Secretory leukocyte protease inhibitor, LUM: Lumican, ATP2C2: ATPase secretory pathway Ca2+ transporting 2, KCNE3: Potassium voltage-gated channel, Isk-related family, member 3, SPAI-2: Sodium/potassium ATPase inhibitor-2, DMC1: Disrupted meiotic cDNA 1. WebGestalt was used to perform analysis of DEGs co-occurrence in the ileum of piglets by comparison of the control and probiotics groups, and identified 10 gene ontologies in the biological process range. Seven out of 10 gene ontologies were significantly associated with immune response.

Additionally, using Innate Immune Database [41], we identified that six genes such as RSAD2, LUM, phospholipase A2 group IIA (PLA2G2A), Olfactomedin-4 (OLFM4), Deleted in malignant brain tumors 1 protein (DMBT1), complement component 6 (C6) among 25 DEGs are associated with mammalian immune systems.

**Fig 7:**
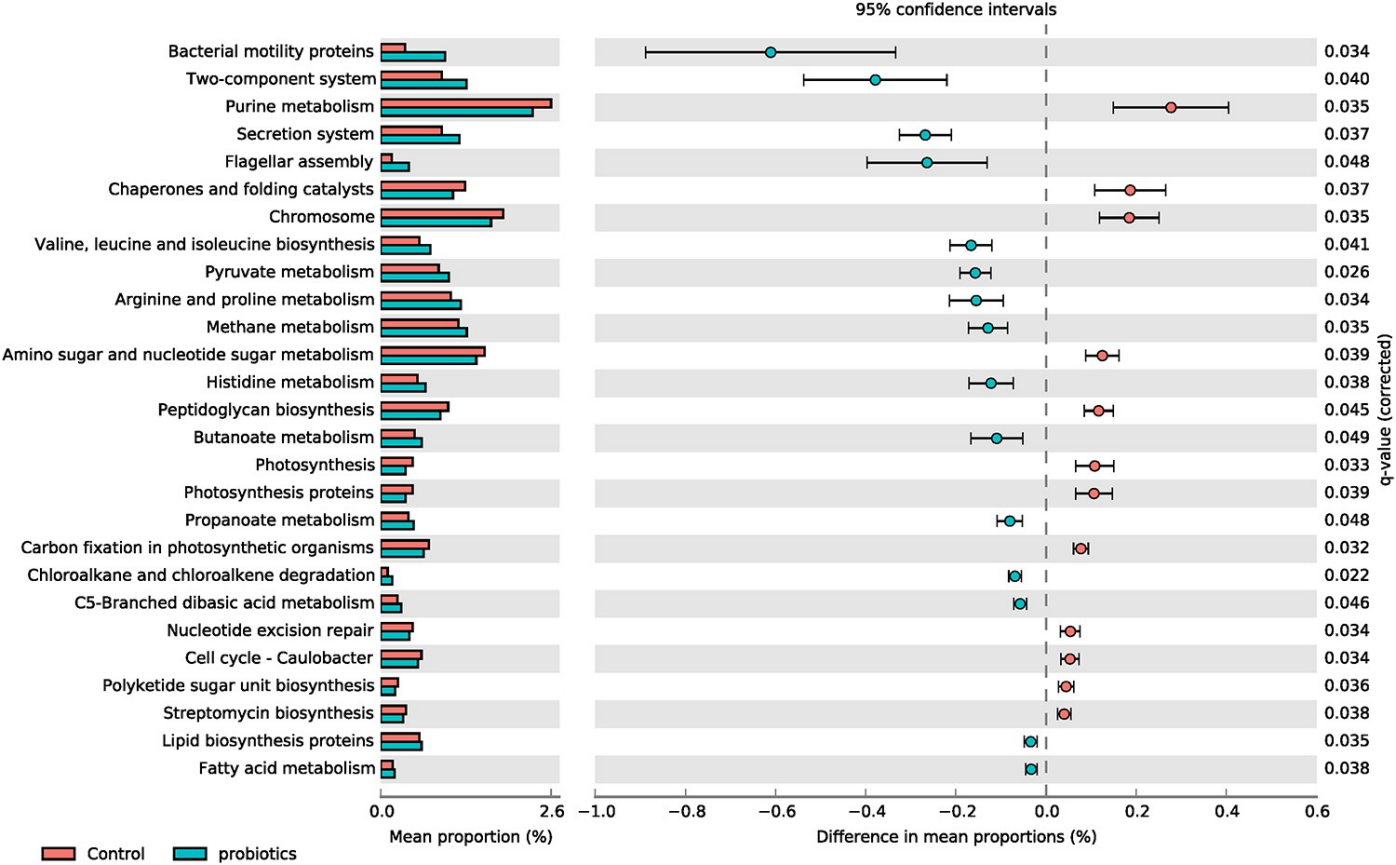
PICRUSt prediction of functional profiling of the microbial communities based on the 16S rRNA gene sequences. A total of 28 KEGG pathways were significantly changed in the probiotics group compared with the control group. Extended error bar plot indicating differences in functional profiles of the control and probiotics microbiota (at taxonomic Level 3). All unclassified reads were removed and q-value greater than 0.05 is displayed. And then, effect size (difference between proportions) was less than 0.03. Categories are sorted by effect sizes calculated using two-sided Welch’s t-test and multiple test correction is Benjamini-Hochberg FDR. Bar plots on the left side displayed the mean proportion of each KEGG pathway. Dot plots on the right show the differences in mean proportions between the two indicated groups using q-values.

We performed PICRUSt to integrate microbiome and RNA-seq data. Through PICRUSt analysis predicting functional profiling of the microbial communities based on the 16S rRNA gene sequences, a total of 28 KEGG pathways were significantly changed in the probiotics group compared with the control group (Fig 7). Bacterial Probiotic supplementation increased the pathways of microbioal structure and cell signalling such as motility proteins, two-component system, secretion system and flagellar assembly. Regarding to microbial metabolism related to gut health, valine, leucine and isoleucine biosynthesis, c5-branched dibasic acid metabolism, and butanoate metabolism were increased in the pbiotics group compared with the control group.

**Fig 8:**
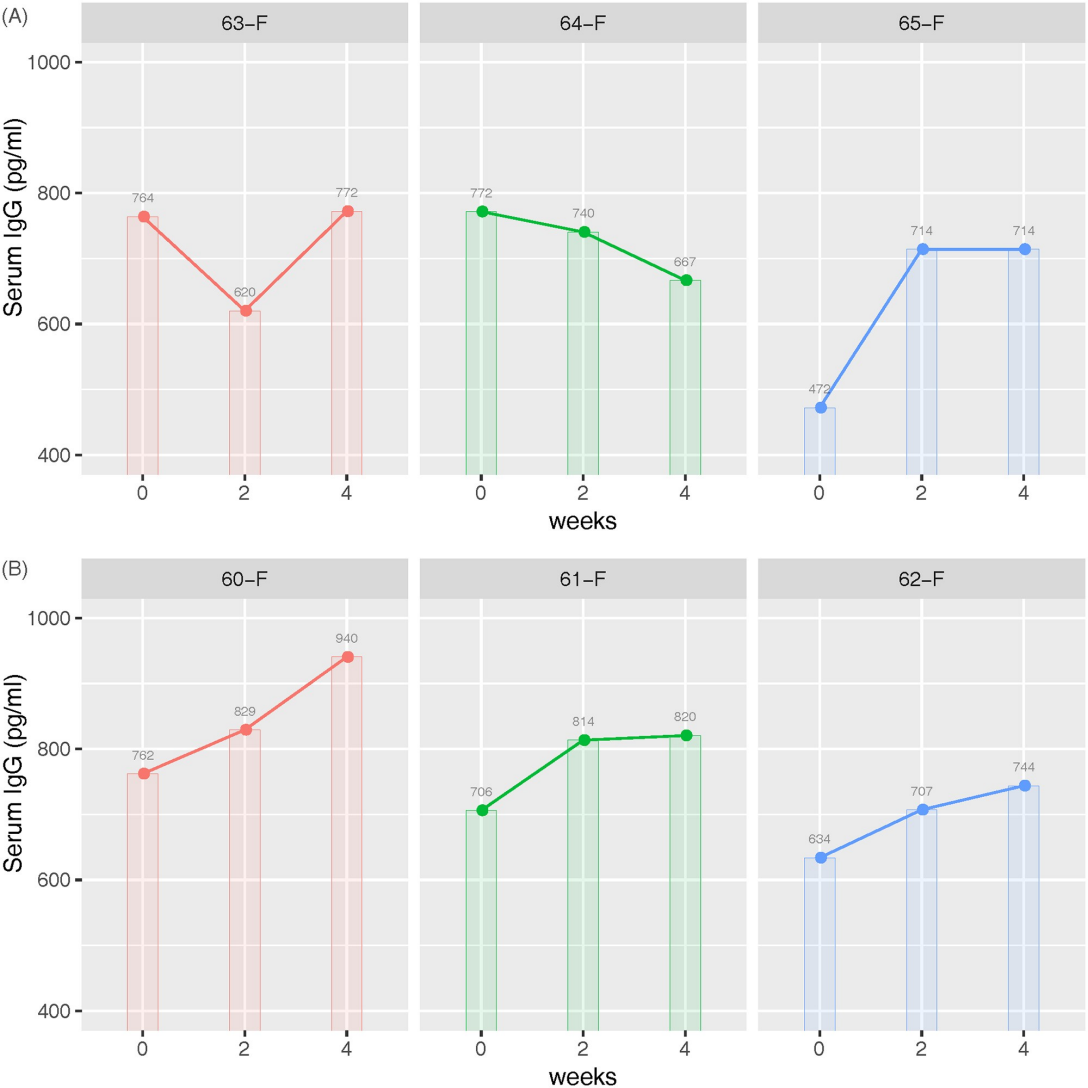
Comparison of serum IgG between the control and probiotics groups. (A) is control group and (B) is probiotics group. Blood samples were collected on days 0, 14 and 28 for measurement of serum immunoglobulin G (IgG) during a 4 week trial. The serum IgG was increased with consistency by time in the probiotics group compared to the control group.

The serum IgG was increased with consistency by time in the probiotics group compared to the control group (Fig 8).

Lastly, we randomly selected six DEGs between the control and probiotics groups and performed qRT-PCR validation for each gene expression. And we could find that RNA sequencing readcount and qRT-PCR expression level of selected six DEGs were highly correlative (S5 Fig).

## Discussion

The weaning of piglets is a big challenge to the developing immune system adapting to gastrointestinal microbial colonization and antigens from milk to feed [22]. Preventing the weaning stress, feed antibiotics have been widely used since early 1950s in the swine industry to promote the growth and prevent infections [42]. However, their side effects including antimicrobial resistance and residues in swine products have caused a major concern for the modern society. Since 2006, the European Union has banned the use of feed antibiotics [43]. As an alternative additive, probiotics have beneficial effects by relieving intestinal microbiota disorders, decreasing intestinal pathogenic bacteria, promoting growth, and improving feed efficiency [44]. *L*. *plantarum* has been recognized as one of the most promising feed probiotics having beneficial effects on the gastrointestinal health and growth of weaned piglets [45]. Through the feeding experiment, dietary supplementation of *L*. *plantarum* in nursery pig diets increased growth performance and blood immunoglobulin concentrations, and improved intestinal morphology and microbiota [46]. However, a lot of gaps remain in the exact roles by probiotics in modulation of piglets’ microbiota and immune response. So we studied to identify the role of probiotics in gut microbiota and immune response of piglets through the analysis of fecal metagenome and intestinal transcriptome. Several studies investigated the metagenomic characterization of the intestinal microbiota using feces and the major role of small intestine to support the body’s immune system. Especially, Peyer’s patches, located within the ileum of the small intestine, are an important part of the digestive tract’s local immune system. The presence of gut flora positively contributes to the host’s immune system [47]. Therefore, according to the purpose of this study, we thought that it was reasonable to analyze the changes of fecal metagenome and the transcriptome of small intestine.

Microbial enumeration of LAB was consistent with the similar result from other researchers [48], who also observed increased LAB complexes in the digestive tract of pigs after probiotic supplementation. Another study in weaned piglets revealed that 0.1% of *Lactobacillus reuteri* and *L*. *plantarum* complex elevated the fecal population of *Lactobacillus* [49]. The microbiome of the intestinal tract undergoes a post-weaning transition where lactobacilli dominate the microbiota of suckling piglets while members of Firmicutes and *Bacteroides* are predominant in adult swine [50, 51]. After weaning, the relative abundancies of lactobacilli begin to wane, paving way for pathogenic microorganisms to colonize the gut. However, supplementation with probiotics has been demonstrated to reduce the numbers of pathogens in the gut while increasing the abundance of commensal bacteria [50, 52].

Photomicrographs of the epithelial layer of intestine resonate with results in another study by [53]. Similarly, Inclusion of probiotic LAB in the diet fed to one-day old broilers increased the villus height in the jejunum [54]. However, a study by [55] showed no effect on villus height and crypt depth in the small intestines of weaned piglets supplemented with probiotics. The important component of the gastrointestinal tract involved in the absorption of nutrients into the body system across the small intestines is the villa [53, 56]. Modulation of the intestinal microbiome ultimately alters the intestinal morphology. The small intestine’s role and influence in digestion is closely related to its mucosal structure especially the villus size and shape [57]. Tthe density and size of villi directly affects the absorption capacity in the intestines [58].

The results of gut microbiota diversity agree with other studies [59–61], and suggest a positive effect conferred by probiotics on swine gut health. During the weaning period, the gut microbiota undergoes dramatic and partly revocable alterations in the first seven days leading to shifts in the intestinal environment [62], hence various studies have indicated that supplementation of probiotics could help to balance the bacterial community in weaned piglets [20, 63]. The gut microbiota influences on gathering, storing and expending of the energy acquired from the diet. Many researchers indicated that the manipulation of gut microbiota could contribute enzymes for digestion and promote weight gain [64]. Commensal microbiota is important in salvaging energy from otherwise indigestible carbohydrates and also confers protection to the host through forming a mucosal defense frontline [65].

The relative abundant patterns of Firmicutes, Bacteroidetes and Spirochaetes in current study agree with others [51, 66]. The population of Bacteroidetes decreased while Firmicutes and Spirochaetes increased in the probiotics group compared to the control group in agreement with the result from [60]. The results of this study reveal that supplementation of probiotics has remarkable alterations on the microbial community in the gut at phylum level, especially on Bacteroidetes, Firmicutes and Spirochaetes. The pig intestinal microbiota has distinctive differences in its composition, however, more than 90% of this microbiota belongs to two major phyla, Firmicutes and Bacteroidetes [67], which is due to the fact that most of the members of these phyla are anaerobic and hence favored by the anaerobic environment in the colon [68]. The ratio of the two phyla in the gut can affect the capacity to absorb nutrients from the ingested feed [69] and thus their modulation in the weaned piglets is of paramount importance. Studies have also shown that two predominant phyla in the gut of human, mouse and swine [70, 71] which have direct correlations to lipid metabolism and thus their manipulation could be an alternative to treatment of obesity [69]. Other studies also showed that body fat in the common pig breeds had close relations with percentages of Bacteroidetes and Firmicutes in the gut microbiota [69]. The percentage compositions of the orders Clostridiales, Erysipelotrichales, Sphaerochaetales and Spirochaetales in the fecal samples of the probiotics group were higher than those of the control at the end of the study while the percentage of Bacteroidales was lower (S3 Table). Results of this study at order level are quite similar with results described by other researchers [66]. The relative abundance of family *Prevotellaceae* occupied the largest part in the control group, but was declined in the probiotics group. However, others statistical significant families (*Spirochaetaceae*, *Eryspelotrichaceae*, *Sphaerochaetaceae* and *Christensenellaceae)* were elevated in the probiotics group. In previous study, *Spirochaetaceae* was demonstrated to have a positive correlation with weight of the host, while *Prevotellaceae* had a negative correlation [72]. Thus manipulating these would positively impact on weight gain in the piglets. Members of the family *Erysipelotrichaceae* were relatively more abundant in the probiotics group (2.85% vs 4.08%). A contrasting result was observed in a study by [73], who found out that relative abundance of *Erysipelotrichaceae* was lower in probiotic supplemented broilers compared to the antibiotic supplemented broilers. These novel findings however point to the fact that modulation of the concentrations of members of this family can be used to substitute antibiotics in feed. Members of *Erysipelotrichaceae* family are highly immunogenic and flourish after treatment with antibiotics [73], and their increased abundance in colonic fecal microbiota has been closely associated with increased dietary fat intake, body weight, fat deposition and reduced fecal short chain fatty acids in mice [74]. We consider that *Ruminococcaceae* was important, because it was the second largest occupation in the control group. Then, the relative abundance of *Ruminococcaceae* was elevated in the probiotics treatment groups (up to 21.8% ~ 25.33%) compared to the control. It was reported that the increase of *Ruminococcaceae* in weaned piglets compared to the nursing period [75]. Members of the family *Ruminococcaceae* have been linked with attributes including; cellulolytic activity and production of SCFAs which have numerable benefits in modulating gut health, inhibition of *Salmonella* growth and having anti-inflammatory effects [76]. The relative abundance of *Lactobacillaceae* in the probiotic group was numerically lower than that in the control group, although it was not statistically different (P = 0.164) Other studies also reported the similar results in the relative abundance of *Lactobacillaceae* and *Lactobacillus* in pigs administrated by *Lactobacillus* spp [77, 78]. The study administrating *L*. *plantarum* PFM105 to weaned piglets showed that the relative abundance of *Lactobacillaceae* of the colonic microbiota was numerically lower in the probiotics group compared to control group [77]. Another study using *L*. *rhamnosus* GG did not detect the statistical difference in the relative abundance of *Lactobacillus* in ileal mucosal microbiota between the control and probiotics groups [78]. This conflicting may result from the limitations of available metagenomic database and prediction tools [79], interfering with the identification of the reads of 16S rRNA originated from pigs. Compensating for these limitations, we performed the enumeration of viable LAB to determine the influence of probiotics on the community of LAB.

The ability of gut microbiota to inhibit colonization of pathogens is mediated through a number of mechanisms, including direct killing, competition for limited nutrients, and enhanced immune responses [80]. Basically, we assumed if piglets continued to consume probiotics, intestine microbial diversity increases, which would be associated with benefits on immune status. Thus, we predicted the expression pattern of DEGs relevant to our presumption. We performed gene ontology analysis (biological process, cellular component and molecular function) to promote biological understanding of 25 DEGs between the control and probiotics groups. Especially, the PLA2G2A genes was the most significant gene in 25 DEGs, and the protein encoded by this gene is a member of the phospholipase A2 family (PLA2), which constitute a diverse family of enzymes with respect to sequence, function, localization, and divalent cation requirements. The gene expression analysis of the IL-22-treated HepG2 cells (human hepatocellular carcinoma line) identified PLA2G2A as an upregulated antimicrobial protein in the pathogen infection study [81]. BPI, antimicrobial protein, plays a significant function in the natural defense of the host organism such as killing gram negative bacteria, neutralizing endotoxin, contributing phagocytosis through complement activation and opsonization, inhibiting angiogenesis and releasing of inflammatory mediators [82]. RSAD2 is stimulated by interferon, involved in innate immunity and contributes primarily to antiviral responses through inhibition of the DNA and RNA viruses’ replication [83, 84]. SLPI extensively inhibits several leukocyte serine proteases [85] and plays several important roles in both normal neutrophil formation and inflammation sites [86]. Cytokine is a diverse intercellular signaling protein that affects several targets, which was associated with an immunologically dependent inflammatory response [87]. LUM encodes a Lumican, an extracellular matrix protein that member of the small leucine-rich proteoglycan (SLRP) family. LUM has emerged as modulators of the inflammatory responses, which alters and impact cytokine expression [88]. OLFM4, is a strongly expressed glycoprotein in the intestine, participating in innate immunity and inflammation, and upregulated by gastrointestinal tract diseases [89]. DMBT1 and C6 are also involved in mucosal innate immunity and inflammatory response [90, 91]. Altogether, transcriptome data in this study presents probiotic supplementation down-regulated most of immune associated genes (BPI, RSAD2, SLPI, LUM, OLFM4, DMBT1 and C6) except PLA2G2A among eight DEGs identified by ontology analysis and Innate Immune DB. These gene expression data imply attenuated inflammation status [92], enhancing the integrity of the intestinal epithelial layers via the development and maintenance of the healthy commensal microbiota represented by diversity and richness [93].

The motility is achieved in most bacterial species by the flagellar apparatus. Traditionally, the flagellum has been regarded only as a motility organelle, but recently it has become evident that flagella have several other biological functions [94]. Especially, from the mammalian host perspective, the flagellum is relevant for immune defense. Additionally, flagella have also been reported to function as adhesion [94]. Bacterial adhesion is a important initial step in bacterial colonization and persistence, both for pathogens and commensals. Two-component signaling cascade involving chemotaxis-related proteins affects flagellar rotation [94]. We thought that these functions could help microbiome settlement and maintenance of effect in the probiotics group. Notably, PICRUSt analysis of microbial communities predicted that probiotic supplemenation increased branched chain amino acid biosynthesis and butyrate metabolism, which may improve the integrity of gut health in weaned piglets. Branched chain amino acids promote intestinal development and maintenance, and immune defense functions [95]. Butyrate is a primary energy source of colonocytes, and involves in various immune responses to prevent inflammation and oxidative stress in colon [96]. Immunoglobulin G (IgG) is the main type of antibody found in blood and extracellular fluid, protecting the body from infection by binding pathogens such as viruses, bacteria and fungi. We expected that the serum IgG is a more direct index to determine the piglet’s health satus than other indices such as average daily gain and feed intake, and measured its concentrations at 2-week intervals in the control and probiotics groups. The data of serrum IgG imply that probiotics may give the positive effect on the piglets’s immune status via regulation of transcriptome in small intestine afftected by microbiome. Through the integration of microbiome data with transcriptome data, we broaden the understanding relationships between intestinal microbiome and host intestine.

## Conclusion

In conclusion, liquid probiotic *L*. *plantarum* JDFM LP11 promoted the integrity of intestinal epithelial layers and serum IgG level in weaned piglets. Probiotic supplementation resulted in higher diversity and richness including dynamic changes of microbial composition in fecal microbiota. Immune associated BPI, RSAD2, SLPI, LUM, OLFM4, DMBT1 and C6 genes were down-regulated by probiotics except PLA2G2A in the ileum of piglets. Increased metabolic pathways of branched chain amino acid biosynthesis and butyrate metabolism predicted from microbial communities imply benefits of probiotics on gut health. Our data suggest roles of probiotics to modify the crosstalk between commensal microbiota and host gut via attenuating the immune associated gene expression towards gut inflammation.

## Supporting information

S1 Fig Enumeration of lactic acid bacteria in fecal samples between the control and probiotics group. Each graph represents the mean±SD of three replicates per group. The number of lactic acid bacteria in fecal samples was significantly higher in the probiotics group compared to the control at p<0.05.
(DOCX)

S2 Fig Histogram representing taxonomic composition and relative abundance (>0.1%) at phylum level in fecal samples between the control and probiotics groups. (DOCX)

S3 Fig Histogram representing taxonomic composition and relative abundance (>0.1%) at class level in fecal samples between the control and probiotics groups. (DOCX)

S4 Fig Histogram representing taxonomic composition and relative abundance (>0.1%) at order level in fecal samples between the control and probiotics groups. (DOCX)

S5 Fig Gene expression in the small intestine (ileum) of piglets between the control and probiotics groups using qRT-PCR. (DOCX)

S6 Fig Rarefaction of the control (63, 64, 65) and probiotics (60, 61, 62) groups. (DOCX)

S1 Table Taxonomic composition and relative abundance at phylum level in fecal samples between the control and probiotics groups. (DOCX)

S2 Table Taxonomic composition and relative abundance at class level in fecal samples between the control and probiotics groups. (DOCX)

S3 Table Taxonomic composition and relative abundance at order level in fecal samples between the control and probiotics groups. (DOCX)

S4 Table Taxonomic composition and relative abundance at family level in fecal samples between the control and probiotics groups. (DOCX)

S5 Table RNA-seq reads and mapping rate of the small intestine samples. (DOCX)

S6 Table Differentially expressed genes in the small intestine (ileum) between control and probiotics groups. (DOCX)

S7 Table List of DEG primers used in qRT-PCR. (DOCX)
