# Supplementary material for: Beneficial roles of probiotics on the modulation of gut microbiota and immune response in pigs
Source: PLoS One. 2019 Aug 28;14(8):e0220843. doi: 10.1371/journal.pone.0220843 (PMC6713323; doi:10.1371/journal.pone.0220843)
Supplement: S1 Fig — Each graph represents the mean±SD of three replicates per group. The number of lactic acid bacteria in fecal samples was significantly higher in the probiotics group compared to the control at p<0.05. (DOCX) [file pone.0220843.s001.docx]

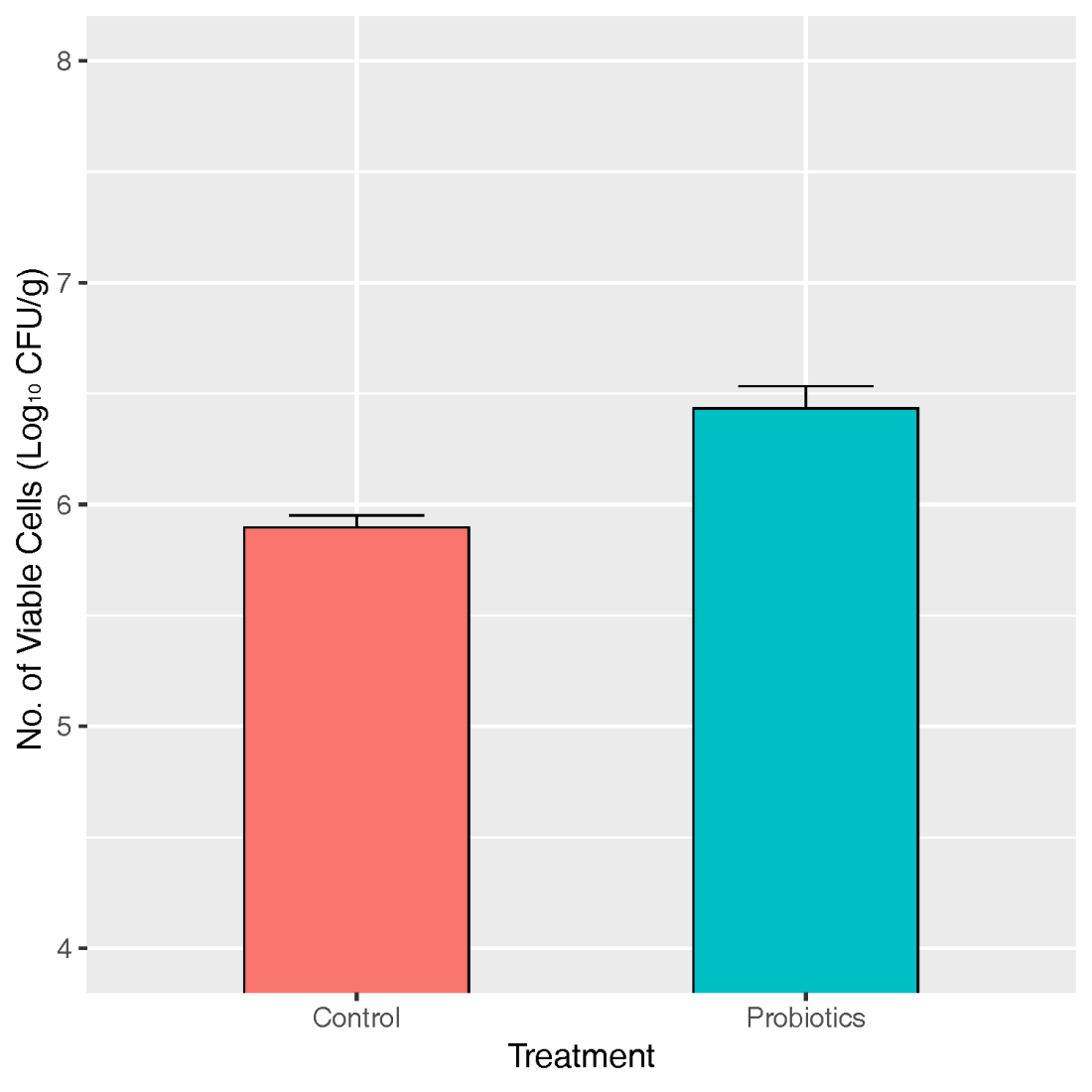


**S1 Fig. Enumeration of Lactic acid bacteria in fecal samples between the control and probiotics groups.** Each graph represents the mean±SD of three replicates per group. The numbers of lactic acid bacteria in fecal samples were significantly higher in the probiotics group compared to the control at p<0.05.
