# Supplementary material for: Beneficial roles of probiotics on the modulation of gut microbiota and immune response in pigs
Source: PLoS One. 2019 Aug 28;14(8):e0220843. doi: 10.1371/journal.pone.0220843 (PMC6713323; doi:10.1371/journal.pone.0220843)
Supplement: S2 Fig — (DOCX) [file pone.0220843.s002.docx]

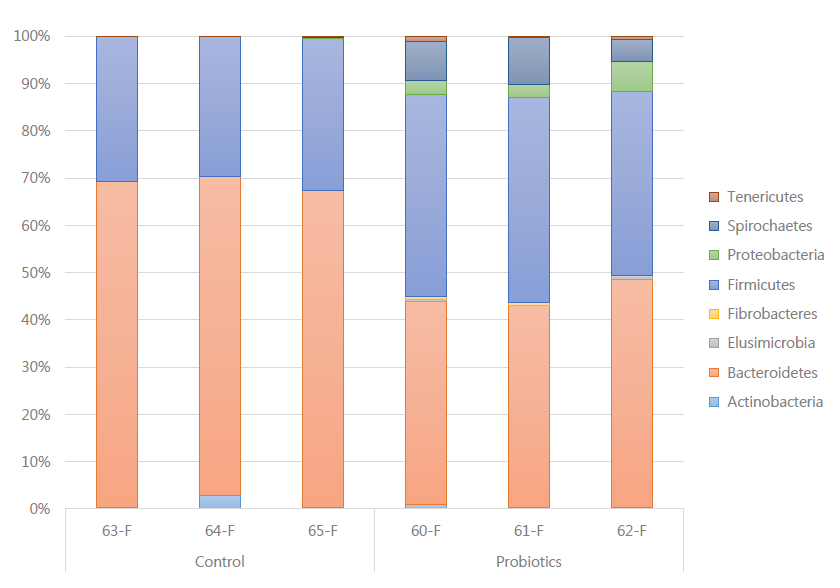


**S2 Fig. Histogram representing taxonomic composition and relative abundance (>0.1%) at phylum level in fecal samples between the control and probiotics groups.**
