# Supplementary material for: Beneficial roles of probiotics on the modulation of gut microbiota and immune response in pigs
Source: PLoS One. 2019 Aug 28;14(8):e0220843. doi: 10.1371/journal.pone.0220843 (PMC6713323; doi:10.1371/journal.pone.0220843)
Supplement: S5 Fig — (DOCX) [file pone.0220843.s005.docx]

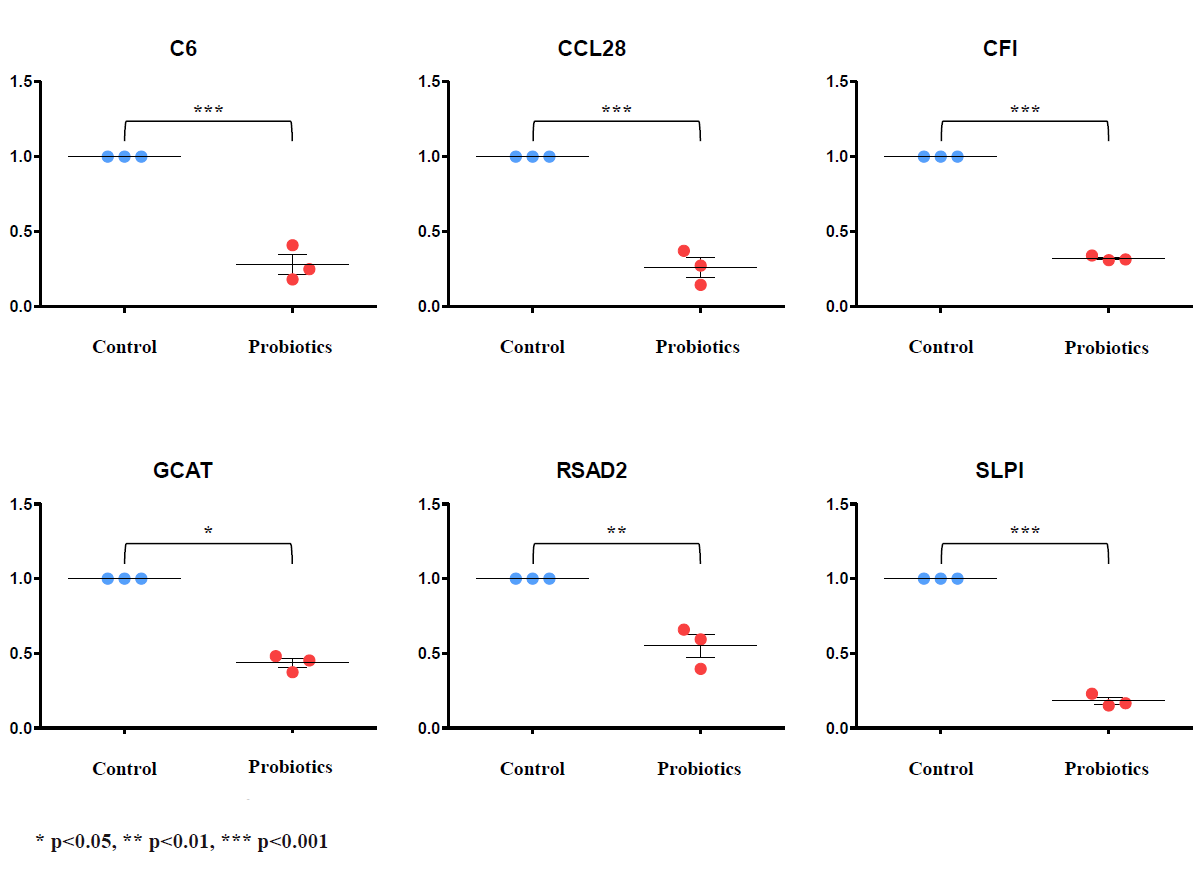


**S5 Fig. Gene expression in the small intestine (ileum) of piglets between the control and probiotics groups using qRT-PCR.**
