# Supplementary figures and images for: Beneficial roles of probiotics on the modulation of gut microbiota and immune response in pigs
Source: PLoS One. 2019 Aug 28;14(8):e0220843. doi: 10.1371/journal.pone.0220843 (PMC6713323; doi:10.1371/journal.pone.0220843)

**
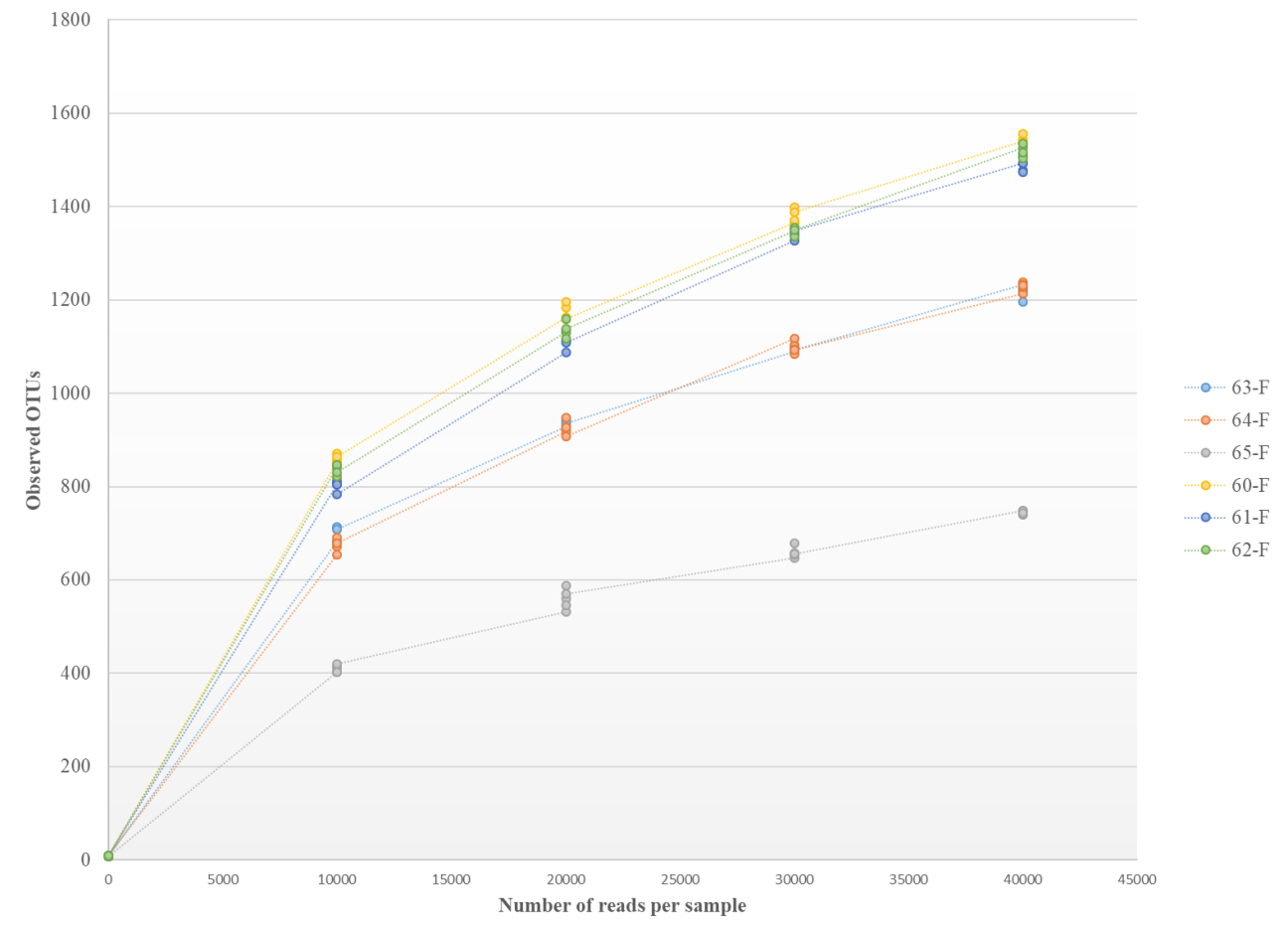
**

**S6 Fig. Rarefaction of the control (63, 64, 65) and probiotics treatment (60, 61, 62) groups.**

Supplement: S6 Fig — (DOCX) [file pone.0220843.s006.docx]
