# Supplementary material for: Beneficial roles of probiotics on the modulation of gut microbiota and immune response in pigs
Source: PLoS One. 2019 Aug 28;14(8):e0220843. doi: 10.1371/journal.pone.0220843 (PMC6713323; doi:10.1371/journal.pone.0220843)
Supplement: S1 Table — (DOCX) [file pone.0220843.s007.docx]

**S1 Table. Taxonomic composition and relative abundance at phylum level in fecal samples between the control and probiotics groups.**

| phylum | Control | | | Probiotics treatment group | | | T-test Pvalue |
| --- | --- | --- | --- | --- | --- | --- | --- |
|  | 63-F | 64-F | 65-F | 60-F | 61-F | 62-F |  |
| Actinobacteria | 0.30% | 2.79% | 0.35% | 0.98% | 0.37% | 0.21% | 0.531 |
| **Bacteroidetes*** | **69.08%** | **67.31%** | **66.99%** | **42.93%** | **42.11%** | **47.83%** | **0.003** |
| Elusimicrobia | 0.00% | 0.00% | 0.00% | 0.37% | 0.00% | 0.91% | 0.247 |
| Fibrobacteres | 0.00% | 0.00% | 0.00% | 0.51% | 0.79% | 0.05% | 0.175 |
| **Firmicutes*** | **30.53%** | **29.49%** | **32.27%** | **42.81%** | **42.87%** | **38.55%** | **0.006** |
| Proteobacteria | 0.01% | 0.05% | 0.26% | 2.95% | 2.69% | 6.24% | 0.077 |
| **Spirochaetes*** | **0.01%** | **0.06%** | **0.13%** | **8.20%** | **9.74%** | **4.64%** | **0.039** |
| Tenericutes | 0.06% | 0.00% | 0.01% | 1.10% | 0.31% | 0.60% | 0.108 |
