# Supplementary material for: Beneficial roles of probiotics on the modulation of gut microbiota and immune response in pigs
Source: PLoS One. 2019 Aug 28;14(8):e0220843. doi: 10.1371/journal.pone.0220843 (PMC6713323; doi:10.1371/journal.pone.0220843)
Supplement: S2 Table — (DOCX) [file pone.0220843.s008.docx]

**S2 Table. Taxonomic composition and relative abundance at class level in fecal samples between the control and probiotics groups.**

| class | Control | | | Probiotics treatment group | | | T-test P-value |
| --- | --- | --- | --- | --- | --- | --- | --- |
|  | 63-F | 64-F | 65-F | 60-F | 61-F | 62-F |  |
| Bacilli | 3.63% | 3.69% | 10.92% | 1.61% | 0.20% | 1.20% | 0.167 |
| **Bacteroidia*** | **69.08%** | **67.31%** | **66.99%** | **42.93%** | **42.11%** | **47.83%** | **0.003** |
| **Clostridia*** | **26.34%** | **24.90%** | **20.52%** | **37.11%** | **39.82%** | **34.43%** | **0.005** |
| Coriobacteriia | 0.27% | 2.77% | 0.32% | 0.96% | 0.35% | 0.19% | 0.536 |
| Deltaproteobacteria | 0.00% | 0.00% | 0.00% | 0.33% | 0.18% | 0.65% | 0.107 |
| Elusimicrobia | 0.00% | 0.00% | 0.00% | 0.37% | 0.00% | 0.91% | 0.247 |
| Epsilonproteobacteria | 0.00% | 0.04% | 0.01% | 0.69% | 1.80% | 2.24% | 0.077 |
| **Erysipelotrichi*** | **0.56%** | **0.89%** | **0.82%** | **4.08%** | **2.85%** | **2.91%** | **0.019** |
| Fibrobacteria | 0.00% | 0.00% | 0.00% | 0.51% | 0.79% | 0.05% | 0.175 |
| Gammaproteobacteria | 0.00% | 0.00% | 0.23% | 1.79% | 0.46% | 3.20% | 0.157 |
| Mollicutes | 0.06% | 0.00% | 0.01% | 1.09% | 0.27% | 0.59% | 0.119 |
| **Spirochaetes*** | **0.01%** | **0.06%** | **0.13%** | **8.20%** | **9.74%** | **4.64%** | **0.039** |
