# Supplementary material for: Beneficial roles of probiotics on the modulation of gut microbiota and immune response in pigs
Source: PLoS One. 2019 Aug 28;14(8):e0220843. doi: 10.1371/journal.pone.0220843 (PMC6713323; doi:10.1371/journal.pone.0220843)
Supplement: S3 Table — (DOCX) [file pone.0220843.s009.docx]

**S3 Table. Taxonomic composition and relative abundance at order level in fecal samples between the control and probiotics groups.**

| order | Control | | | Probiotics treatment group | | | T-test  P-value |
| --- | --- | --- | --- | --- | --- | --- | --- |
|  | 63-F | 64-F | 65-F | 60-F | 61-F | 62-F |  |
| Aeromonadales | 0.00% | 0.00% | 0.22% | 1.76% | 0.44% | 2.94% | 0.150 |
| **Bacteroidales*** | **69.08%** | **67.31%** | **66.99%** | **42.93%** | **42.11%** | **47.83%** | **0.003** |
| Campylobacterales | 0.00% | 0.04% | 0.01% | 0.69% | 1.80% | 2.24% | 0.077 |
| **Clostridiales*** | **26.34%** | **24.90%** | **20.52%** | **37.11%** | **39.82%** | **34.43%** | **0.005** |
| Coriobacteriales | 0.27% | 2.77% | 0.32% | 0.96% | 0.35% | 0.19% | 0.536 |
| Desulfovibrionales | 0.00% | 0.00% | 0.00% | 0.32% | 0.11% | 0.59% | 0.136 |
| Elusimicrobiales | 0.00% | 0.00% | 0.00% | 0.37% | 0.00% | 0.91% | 0.247 |
| **Erysipelotrichales*** | **0.56%** | **0.89%** | **0.82%** | **4.08%** | **2.85%** | **2.91%** | **0.019** |
| Fibrobacterales | 0.00% | 0.00% | 0.00% | 0.51% | 0.79% | 0.05% | 0.175 |
| Lactobacillales | 3.57% | 3.60% | 10.90% | 1.61% | 0.20% | 1.20% | 0.172 |
| RF39 | 0.05% | 0.00% | 0.01% | 1.09% | 0.27% | 0.59% | 0.118 |
| **Sphaerochaetales*** | **0.00%** | **0.06%** | **0.12%** | **0.20%** | **0.36%** | **0.28%** | **0.023** |
| **Spirochaetales*** | **0.00%** | **0.00%** | **0.00%** | **8.00%** | **9.38%** | **4.35%** | **0.040** |
