# Supplementary material for: Beneficial roles of probiotics on the modulation of gut microbiota and immune response in pigs
Source: PLoS One. 2019 Aug 28;14(8):e0220843. doi: 10.1371/journal.pone.0220843 (PMC6713323; doi:10.1371/journal.pone.0220843)
Supplement: S4 Table — (DOCX) [file pone.0220843.s010.docx]

**S4 Table. Taxonomic composition and relative abundance at family level in fecal samples between the control and probiotics groups.**

| family | Control | | | Probiotics treatment group | | | T-test Pvalue |
| --- | --- | --- | --- | --- | --- | --- | --- |
|  | 63-F | 64-F | 65-F | 60-F | 61-F | 62-F |  |
| Bacteroidaceae | 0.05% | 0.00% | 0.07% | 0.20% | 1.35% | 0.07% | 0.346 |
| Campylobacteraceae | 0.00% | 0.00% | 0.00% | 0.69% | 1.78% | 2.15% | 0.073 |
| **Christensenellaceae*** | **0.00%** | **0.00%** | **0.00%** | **0.31%** | **0.42%** | **0.45%** | **0.012** |
| Clostridiaceae | 1.15% | 0.52% | 1.21% | 1.70% | 1.44% | 3.03% | 0.142 |
| Coriobacteriaceae | 0.27% | 2.77% | 0.32% | 0.96% | 0.35% | 0.19% | 0.536 |
| Desulfovibrionaceae | 0.00% | 0.00% | 0.00% | 0.32% | 0.11% | 0.59% | 0.136 |
| Elusimicrobiaceae | 0.00% | 0.00% | 0.00% | 0.37% | 0.00% | 0.91% | 0.247 |
| **Erysipelotrichaceae*** | **0.56%** | **0.89%** | **0.82%** | **4.08%** | **2.85%** | **2.92%** | **0.019** |
| Fibrobacteraceae | 0.00% | 0.00% | 0.00% | 0.51% | 0.79% | 0.05% | 0.175 |
| Lachnospiraceae | 7.86% | 7.73% | 2.72% | 2.77% | 3.27% | 2.80% | 0.202 |
| Lactobacillaceae | 3.49% | 3.55% | 10.79% | 1.59% | 0.08% | 0.75% | 0.164 |
| Porphyromonadaceae | 0.21% | 1.00% | 0.74% | 2.70% | 8.94% | 1.82% | 0.228 |
| **Prevotellaceae*** | **58.37%** | **52.39%** | **51.28%** | **14.53%** | **15.05%** | **15.92%** | **0.002** |
| Ruminococcaceae | 14.63% | 14.42% | 4.41% | 25.33% | 22.18% | 21.80% | 0.059 |
| S24-7 | 2.11% | 6.60% | 9.41% | 7.43% | 2.36% | 14.04% | 0.663 |
| **Sphaerochaetaceae*** | **0.00%** | **0.06%** | **0.12%** | **0.20%** | **0.36%** | **0.28%** | **0.023** |
| **Spirochaetaceae*** | **0.00%** | **0.00%** | **0.00%** | **8.00%** | **9.38%** | **4.36%** | **0.040** |
| Succinivibrionaceae | 0.00% | 0.00% | 0.22% | 1.76% | 0.44% | 2.94% | 0.150 |
| Veillonellaceae | 1.56% | 0.86% | 11.93% | 2.69% | 2.11% | 2.93% | 0.600 |
