# Supplementary material for: Beneficial roles of probiotics on the modulation of gut microbiota and immune response in pigs
Source: PLoS One. 2019 Aug 28;14(8):e0220843. doi: 10.1371/journal.pone.0220843 (PMC6713323; doi:10.1371/journal.pone.0220843)
Supplement: S5 Table — (DOCX) [file pone.0220843.s011.docx]

**S5 Table. RNA-seq reads and mapping rate of the small intestine samples.**

| Sampe ID | Read Order | Yield (bases) | # Reads | Passed Trimmomatic | Overall alignment rate |
| --- | --- | --- | --- | --- | --- |
| 63-F | 1 | 1,158,000,000 | 15,231,985 | 14,493,162 (95.15%) | 97.57% |
|  | 2 | 1,158,000,000 | 15,231,985 | 14,493,162 (95.15%) |  |
| 64-F | 1 | 1,340,500,000 | 17,631,955 | 16,823,411 (95.41%) | 96.92% |
|  | 2 | 1,340,500,000 | 17,631,955 | 16,823,411 (95.41%) |  |
| 65-F | 1 | 1,282,500,000 | 16,878,993 | 16,128,617 (95.55%) | 96.98% |
|  | 2 | 1,282,500,000 | 16,878,993 | 16,128,617 (95.55%) |  |
| 60-F | 1 | 1,285,000,000 | 16,906,796 | 15,961,340 (94.41%) | 97.17% |
|  | 2 | 1,285,000,000 | 16,906,796 | 15,961,340 (94.41%) |  |
| 61-F | 1 | 1,148,000,000 | 15,103,929 | 14,336,923 (94.92%) | 96.83% |
|  | 2 | 1,148,000,000 | 15,103,929 | 14,336,923 (94.92%) |  |
| 62-F | 1 | 1,190,000,000 | 15,652,117 | 14,938,763 (95.44%) | 97.15% |
