# Supplementary material for: Beneficial roles of probiotics on the modulation of gut microbiota and immune response in pigs
Source: PLoS One. 2019 Aug 28;14(8):e0220843. doi: 10.1371/journal.pone.0220843 (PMC6713323; doi:10.1371/journal.pone.0220843)
Supplement: S6 Table — (DOCX) [file pone.0220843.s012.docx]

**S6 Table. Differentially expressed genes in the small intestine (ileum) between control and probiotic groups.**

| Ensemble Gene ID | Gene name | CHR | Gene start (bp) | Gene end (bp) | baseMean | log2FoldChange | lfcSE | P-value | FDR |
| --- | --- | --- | --- | --- | --- | --- | --- | --- | --- |
| ENSSSCG00000004013 | SMOC2 | 1 | 1,193,167 | 1,332,694 | 339.60 | -1.50 | 0.464 | 4.29.E-05 | 1.62.E-02 |
| ENSSSCG00000008648 | RSAD2 | 3 | 128,880,859 | 128,898,072 | 588.35 | -1.75 | 0.550 | 5.02.E-05 | 1.77.E-02 |
| ENSSSCG00000000103 | DMC1 | 5 | 9,433,058 | 9,470,549 | 59.54 | 3.58 | 1.310 | 1.76.E-04 | 3.76.E-02 |
| ENSSSCG00000029945 | GCAT | 5 | 10,031,225 | 10,038,476 | 78.36 | -1.90 | 0.525 | 1.02.E-05 | 7.52.E-03 |
| ENSSSCG00000000916 | LUM | 5 | 91,748,494 | 91,760,563 | 292.18 | -1.73 | 0.560 | 6.42.E-05 | 2.02.E-02 |
| ENSSSCG00000002671 | ATP2C2 | 6 | 4,240,668 | 4,313,279 | 288.37 | -1.74 | 0.381 | 2.18.E-07 | 5.49.E-04 |
| ENSSSCG00000002737 | CHST4 | 6 | 14,535,888 | 14,546,695 | 496.28 | -1.64 | 0.410 | 2.38.E-06 | 3.28.E-03 |
| ENSSSCG00000003494 | PLA2G2A | 6 | 78,327,319 | 78,330,720 | 139.59 | 11.53 | 3.087 | 1.78.E-14 | 2.70.E-10 |
| ENSSSCG00000001441 | - | 7 | 24,274,149 | 24,286,960 | 227.65 | -1.72 | 0.579 | 9.77.E-05 | 2.65.E-02 |
| ENSSSCG00000009138 | CFI | 8 | 112,433,640 | 112,470,946 | 4291.22 | -1.77 | 0.533 | 3.08.E-05 | 1.37.E-02 |
| ENSSSCG00000009182 | - | 8 | 121,020,910 | 121,109,097 | 431.01 | 2.18 | 0.696 | 5.89.E-05 | 2.02.E-02 |
| ENSSSCG00000029323 | KCNE3 | 9 | 8,810,703 | 8,822,341 | 249.51 | -1.56 | 0.433 | 1.20.E-05 | 7.65.E-03 |
| ENSSSCG00000014985 | MMP3 | 9 | 33,410,143 | 33,454,194 | 894.49 | -2.00 | 0.669 | 8.77.E-05 | 2.55.E-02 |
| ENSSSCG00000015086 | TMPRSS4 | 9 | 45,400,130 | 45,435,918 | 50.92 | -1.71 | 0.469 | 1.02.E-05 | 7.52.E-03 |
| ENSSSCG00000028135 | PRTFDC1 | 10 | 50,365,903 | 50,458,591 | 226.23 | -1.54 | 0.394 | 3.66.E-06 | 4.26.E-03 |
| ENSSSCG00000009444 | OLFM4 | 11 | 26,350,032 | 26,375,740 | 6795.24 | -1.65 | 0.573 | 1.26.E-04 | 2.98.E-02 |
| ENSSSCG00000002981 | DMBT1 | 14 | 132,187,148 | 132,230,899 | 1316.97 | -1.97 | 0.553 | 1.26.E-05 | 7.65.E-03 |
| ENSSSCG00000016861 | C6 | 16 | 26,023,986 | 26,083,742 | 1200.64 | -2.03 | 0.622 | 3.48.E-05 | 1.46.E-02 |
| ENSSSCG00000016871 | CCL28 | 16 | 27,989,692 | 28,010,999 | 90.88 | -2.13 | 0.725 | 9.69.E-05 | 2.65.E-02 |
| ENSSSCG00000016941 | RNF180 | 16 | 42,686,965 | 42,952,053 | 314.18 | -1.59 | 0.601 | 2.41.E-04 | 4.68.E-02 |
| ENSSSCG00000026605 | BPI | 17 | 41,352,116 | 41,397,998 | 129.02 | -2.61 | 0.490 | 4.20.E-09 | 1.59.E-05 |
| ENSSSCG00000033675 | SPAI-2 | 17 | 47,541,160 | 47,546,190 | 39072.81 | -1.61 | 0.463 | 1.88.E-05 | 9.84.E-03 |
| ENSSSCG00000022258 | - | 17 | 47,579,012 | 47,583,115 | 392.08 | -2.10 | 0.571 | 8.53.E-06 | 7.28.E-03 |
| ENSSSCG00000039573 | SLPI | 17 | 47,595,158 | 47,599,301 | 438.81 | -2.43 | 0.416 | 2.37.E-10 | 1.80.E-06 |
| ENSSSCG00000031855 | CELF2 | AEMK02000574.1 | 21,398 | 40,449 | 135.93 | 2.77 | 0.775 | 1.26.E-05 | 7.65.E-03 |
