# Supplementary material for: Beneficial roles of probiotics on the modulation of gut microbiota and immune response in pigs
Source: PLoS One. 2019 Aug 28;14(8):e0220843. doi: 10.1371/journal.pone.0220843 (PMC6713323; doi:10.1371/journal.pone.0220843)
Supplement: S7 Table — (DOCX) [file pone.0220843.s013.docx]

**S7 Table. List of DEG primers used in qRT-PCR.**

| Gene | Forward primer sequence (5’ -> 3’) | Reverse primer sequence (5’ -> 3’) |
| --- | --- | --- |
| GAPDH | CTCAACGACCACTTCGTCAA | TCCAGGGGCTCTTACTCCTT |
| C6 | TGGCTACCAGTACTTCAGATGC | CTGGCAAGAGGCAGTCAGTC |
| CCL28 | GAGAGCGGATGGGGATTGTG | TGATTGTGAGGGCTGACACAG |
| CFI | TGGAGTTAAAAGCAGCACGC | AGCAGTCAAAATCCAACAGCC |
| GCAT | CCATCTCAGGAGCCAATCACC | CGGGGTAGCTGAACCCGATG |
| RSAD2 | GTGTCAGCATCGTGAGCAAC | TGACCACGGCCAATAAGGAC |
| SLPI | CAAGTGCACAAGTGACTGGC | GGCCATAGACCACTGGACAC |
